# *VcRR2* regulates chilling-mediated flowering through expression of hormone genes in a transgenic blueberry mutant

**DOI:** 10.1038/s41438-019-0180-0

**Published:** 2019-08-21

**Authors:** Tianyi Lin, Aaron Walworth, Xiaojuan Zong, Gharbia H. Danial, Elise M. Tomaszewski, Pete Callow, Xiumei Han, L. Irina Zaharia, Patrick P. Edger, Gan-yuan Zhong, Guo-qing Song

**Affiliations:** 1 Plant Biotechnology Resource and Outreach Center, Department of Horticulture, Michigan State University, East Lansing, MI 48824 USA; 2 Department of Horticulture, Michigan State University, East Lansing, MI 48824 USA; 3 Aquatic and Crop Resource Development, National Research Council of Canada, Saskatoon, SK S7N 0W9 Canada; 4 Grape Genetics Research Unit, USDA-ARS, Geneva, NY 14456 USA

**Keywords:** Vernalization, Gibberellins

## Abstract

The molecular mechanism underlying dormancy release and the induction of flowering remains poorly understood in woody plants. Mu-legacy is a valuable blueberry mutant, in which a transgene insertion caused increased expression of a *RESPONSE REGULATOR 2*-like gene (*VcRR2*). Mu-legacy plants, compared with nontransgenic ‘Legacy’ plants, show dwarfing, promotion of flower bud formation, and can flower under nonchilling conditions. We conducted transcriptomic comparisons in leaves, chilled and nonchilled flowering buds, and late-pink buds, and analyzed a total of 41 metabolites of six groups of hormones in leaf tissues of both Mu-legacy and ‘Legacy’ plants. These analyses uncovered that increased *VcRR2* expression promotes the expression of a homolog of *Arabidopsis thaliana* ENT-COPALYL DIPHOSPHATE SYNTHETASE 1 (*VcGA1*), which induces new homeostasis of hormones, including increased gibberellin 4 (GA4) levels in Mu-legacy leaves. Consequently, increased expression of *VcRR2* and *VcGA1*, which function in cytokinin responses and gibberellin synthesis, respectively, initiated the reduction in plant height and the enhancement of flower bud formation of the Mu-legacy plants through interactions of multiple approaches. In nonchilled flower buds, 29 differentially expressed transcripts of 17 genes of five groups of hormones were identified in transcriptome comparisons between Mu-legacy and ‘Legacy’ plants, of which 22 were chilling responsive. Thus, these analyses suggest that increased expression of *VcRR2* was collectively responsible for promoting flower bud formation in highbush blueberry under nonchilling conditions. We report here for the first time the importance of *VcRR2* to induce a suite of downstream hormones that promote flowering in woody plants.

## Introduction

Deciduous woody plants have evolved to be winter dormancy-dependent for survival under freezing temperatures and require accumulation of a minimum level of chilling (chilling requirement) to allow resumption of normal growing and flowering in coming seasons^1–4^. Photoperiod and temperature are the major environmental factors in inducing and maintaining dormancy^1,3,5^. Insufficient chill can reduce fruit production by reducing bud break and lessening flower quality^6^. With climate changes, declining chilling has been occurring for decades and is predicted to continue to do so; and consequently, insufficient winter chill has been recognized as a potential limiting factor on fruit production for both wild and cultivated species^7–9^.

Flowering is a prerequisite for fruiting and plays a significant role in the life cycle of angiosperms^4,10^. Vernalization/chilling is necessary for normal flowering of winter-annual and deciduous perennial plants, and several critical genes controlling the process have well been characterized in annual species^4^. For example, *FLOWERING LOCUS C* (*FLC*), a MADS-BOX gene, plays a central role in the vernalization pathway of winter ecotypes of annual *A. thaliana* and is a critical component of the vernalization regulatory loop of *FRIGIDA* (*FRI*)-*FLC-FT* (*FLOWERING LOCUS T*), which seems being conserved in the family *Brassicaceae*^10–12^. Similarly, in vernalization-requiring wheat and barley, a *VRN1–VRN2*–*TaFT* (*VRN3*) loop regulates vernalization-mediated flowering^13^. In parallel, a cluster of six MADS-box transcription factors identified through forward genetics, analogous to *FLC* in *A. thaliana* and *VRN2* in cereals and termed as *DORMANCY-ASSOCIATED MADS-BOX* (*DAM*), are involved in dormancy regulation in peach (*Prunus persica*)^14,15^. These *DAM* genes show high similarities to *A. thaliana AGAMOUS-LIKE 24* (*AGL24*) and *SHORT VEGETATIVE PHASE* (*SVP*) genes^16–18^. Functional analysis of the *DAMs* through reverse genetics has not been reported in peach. To date, in woody plants, there remains a lack of understanding of the physiological, molecular, and genetical basis of chilling-regulated flowering^7^.

Highbush blueberry (*Vaccinium corymbosum* L.) is a major cultivated *Vaccinium* crop in the family *Ericaceae* (Syn. Heath) containing ∼450 species^19,20^. As a perennial shrub, blueberry floral bud initiation often starts before endodormancy^21^. Enough chilling accumulation during endodormancy, ranging from 150 to 1500 chill units, is required for breaking dormancy, and insufficient chilling prevents normal bud break and leads to reduced blueberry production^20^. Compared with annual and other woody species, the knowledge of genetic and molecular control of chilling requirements for flowering is very limited in blueberry. Our recent comparative transcriptome analysis of nonchilled, chilled, and late-pink bud revealed that the orthologs of many well-known flowering pathway genes in annual species, such as *FT, PROTEIN FD (FD), TERMINAL FLOWER 1 (TFL1)*, and *LEAFY* (*LFY)*, MADS-box genes, and hormone genes, were involved in chilling-mediated blueberry bud break in blueberry^22^. However, like what was noted in other woody plants, functional *FLC* orthologs were not found in blueberries^22–24^.

Hormone genes also played roles in blueberry flowering^23,24^. Overexpression of a blueberry *FT* (*VcFT*: hereafter, *Vc* included in front of any gene refers to the ortholog of the gene in blueberry) induced differential expression of a group of hormone pathway genes in transgenic blueberry leaves^23^. Recently, we identified a unique transgenic blueberry event (line) in the background of the cultivar ‘Legacy’ and this line, named as Mu-legacy, was found to be a valuable genetic stock for studying chilling-induced flowering^25^. Unlike nontransgenic ‘Legacy’ and other transgenic events, Mu-legacy and its T_1_ transgenic plants had early flower bud formation and were able to flower under nonchilling conditions. Our initial characterization of this mutant, compared with the nontransgenic control and other transgenic events, revealed several hormone-related pathway genes DE and concluded that hormone changes were likely responsible for the phenotypic changes in Mu-legacy^25^. This was consistent with our previous work that hormones, at least at transcript levels, might function as physiological signals and play a significant role in regulating blueberry flowering, especially chilling-mediated flowering^22,23,25,26^.

To reveal the potential roles of *VcRR2* and hormone(s) in chilling-mediated blueberry flowering, further analysis of the Mu-legacy was conducted in this study through hormone measurements and RNA sequencing. We revealed profiles of both hormones and DE transcripts (DETs) in leaf and bud tissues of the Mu-legacy plants. We found that the decreased auxin and the increased gibberellin 4 (GA4) contents in Mu-legacy leaves likely contributed to plant dwarfing, and promoted flower bud formation and nonchilled flower bud break of the Mu-legacy plants. The Mu-legacy plants provided evidence to show that an increased expression of *VcRR2* played a critical role in regulating blueberry flower bud formation and bud break mainly through hormone pathway genes but not flowering pathway genes. The results provided further evidence that hormones may play critical roles in chilling-mediated flowering in blueberry and possible other woody plants.

## Results

### Flowering behaviors of Mu-legacy plants

**Fig. 1:**
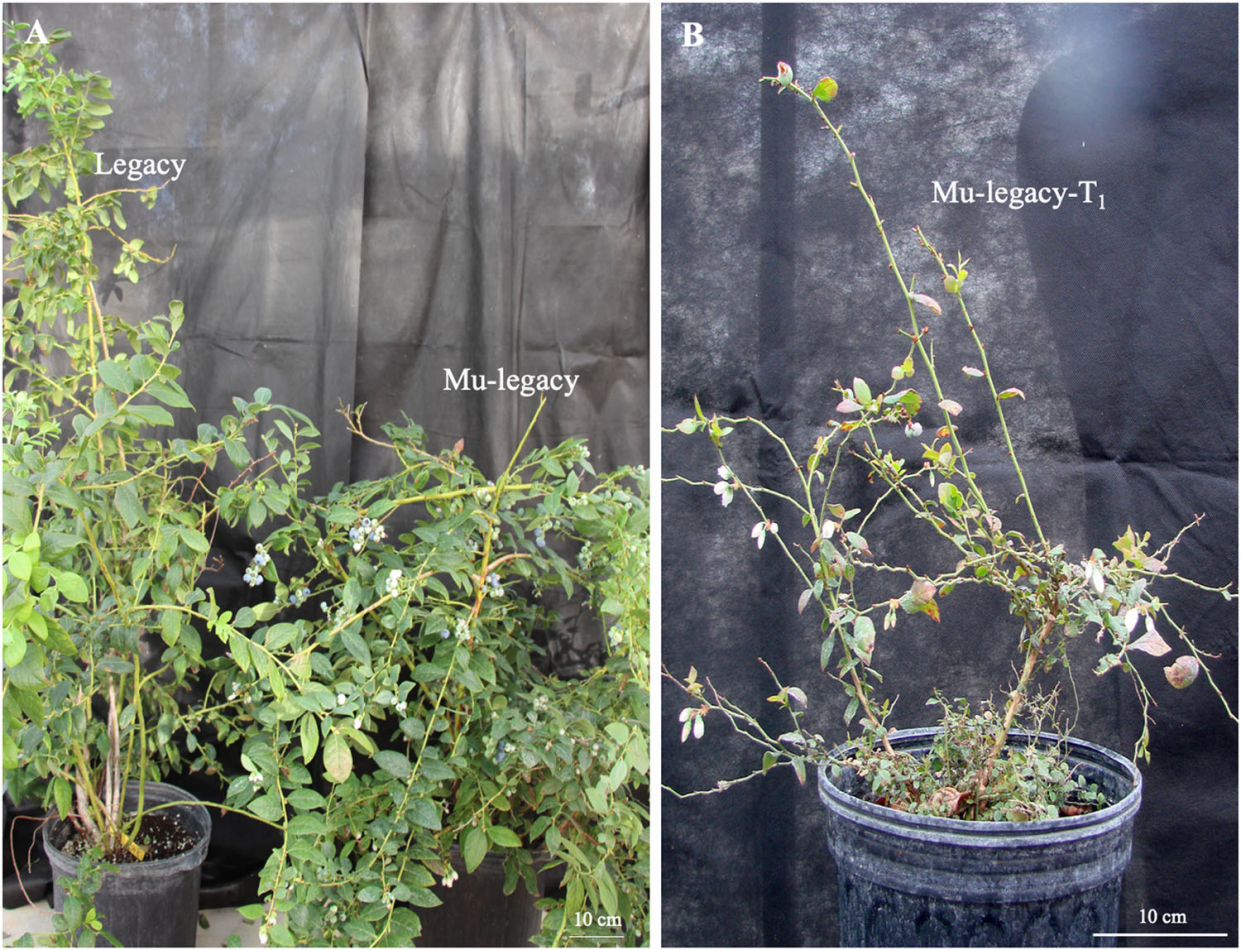
Flowering of ‘Legacy’, Mu-legacy, and Mu-legacy-T_1_ plants under nonchilling conditions. **a** Six-year-old Mu-legacy and ‘Legacy’ plants in October 2017. **b** One of the six 4-year-old Mu-legacy-T_1_ plants in October 2016. No flower bud break was observed in the ‘Legacy’ plant; in contrast, for both Mu-legacy and Mu-legacy-T_1_ plants, flowers and fruits were observed

We exanimated the flowering behaviors of nonchilled 6-year-old ‘Legacy’, Legacy–VcDDF1-OX, Mu-legacy, and Mu-legacy-T_1_ plants. Under the greenhouse conditions (the lowest temperature >20 °C, natural light condition), all the Mu-legacy and the Mu-legacy-T_1_ plants, in contrast to the ‘Legacy’ plants, showed a new break of some flower buds in mid-October of 2017 (Fig. 1); leaf tissues at this developmental stage for both the ‘Legacy’ and the Mu-legacy plants were collected for transcriptome and hormone analyses. The results are consistent with our previous observation^25^. The continuous flowering of the Mu-legacy plants lasted for about 9 months (from October to June)^25^, suggesting that the transgenes and insertion position in the Mu-legacy are responsible for the reduced plant size, the promoted flower bud formation, and the reduced chilling requirement for flowering. Interestingly, the observed plant dwarfing is similar to a typical phenotype of GA deficiency in *Arabidopsis*^27^, while the promoted flower bud formation and reduced chilling requirement for flowering are similar to the phenotypic changes of early flowering and seed dormancy induced by GA-overproduction phenotypes in *Arabidopsis*^28^.

### Contents of major hormones in Mu-legacy leaves

Compared with ‘Legacy’, Mu-legacy carries a transgenic, overexpressed blueberry *DWARF AND DELAYED FLOWERING 1* (*DDF1*) (*VcDDF1*), as well as a mutated and overexpressed blueberry *RESPONSE REGULATOR 2*-like gene (*VcRR2*), which accidently resulted from the position effect of the introduction of the transgene *VcDDF1*^25^. In *A. thaliana*, the *DDF1* activates GA2OX7 by binding to the DRE-like motifs (GCCGAC and ATCGAC) of the GA2OX7’s promoter^29^. The GA2OX7 catalyzes the decay of active GAs^29–33^. On the other hand, *ARABIDOPSIS RESPONSE REGULATOR* 2 gene (*ARR2*) is responsible for cytokinin responses in the cytokinin signaling pathway^34,35^. Since both *VcDDF1* and *VcRR2* are related to hormone signaling, we expect that overexpression of these two genes, especially the *VcDDF1*, would change the hormone profiles of the Mu-legacy plants.

**Fig. 2:**
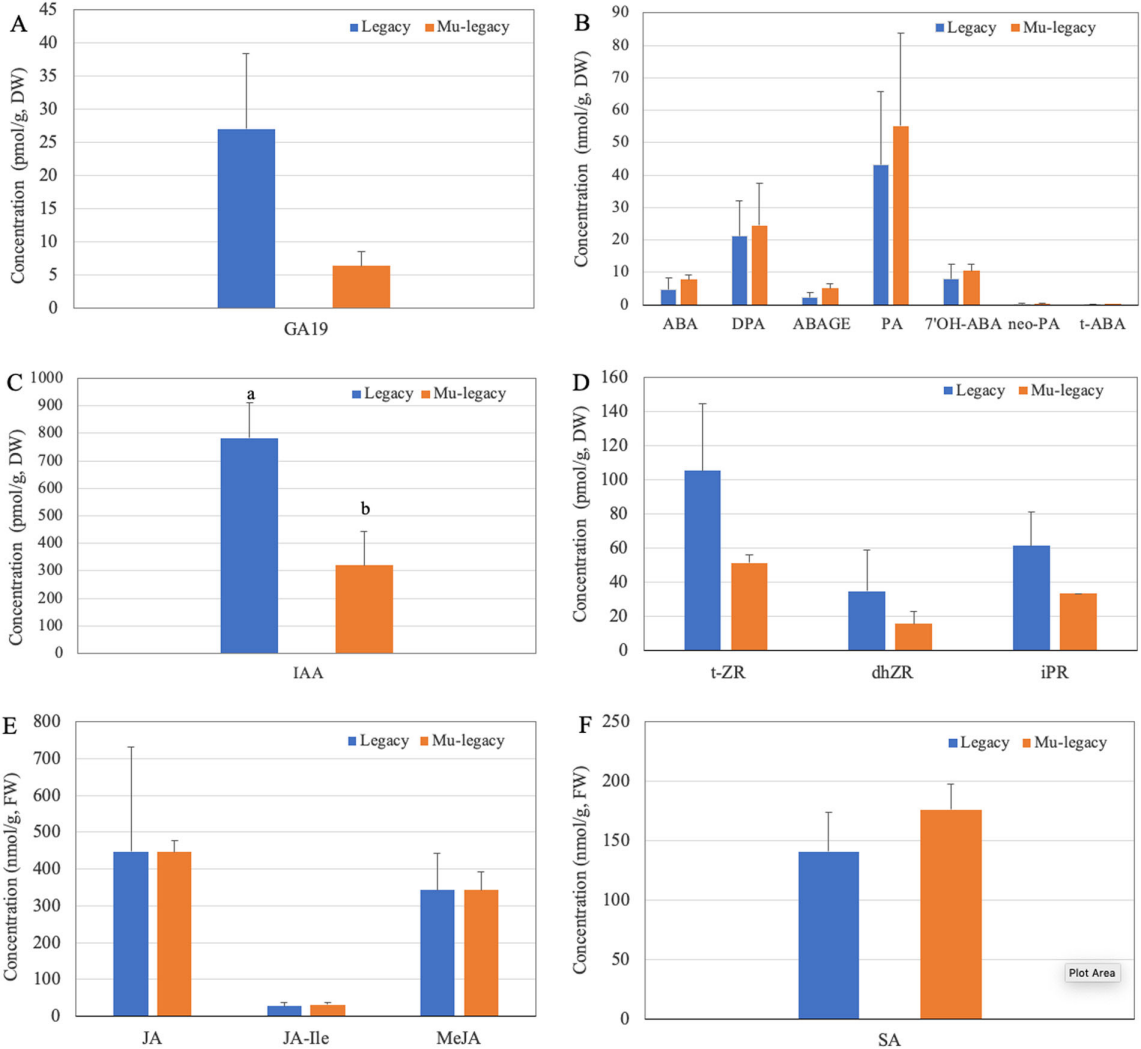
Detected hormones and their concentrations measured in the ‘Legacy’ and Mu-legacy leaves. **a** Gibberellin. **b** ABA and ABA metabolites. **c** Auxin. **d** Cytokinin and cytokinin metabolites. **e** JA and JA metabolites. **f** Salicylic acid. DW: dry weight. FW: fresh weight. Different letters represent a significant difference at *P* < 0.05 between ‘Legacy’ and Mu-legacy leaves. The bars without letters represent no significant differences at *P* < 0.05. GA19: Gibberellin 19. ABA: *cis*-Abscisic acid. DPA: dihydrophaseic acid. ABAGE: Abscisic acid glucose ester. PA: Phaseic acid. 7′OH-ABA: 7′-Hydroxy-abscisic acid. neo-PA: neo-Phaseic acid. t-ABA: trans-Abscisic acid. IAA: Indole-3-acetic acid. t-ZR: (*trans*) Zeatin riboside. dhZR: Dihydrozeatin riboside. IPR: Isopentenyladenosine. MeJA: Methyl jasmonate, JA-Ile: jasmonoyl isoleucine

A total of 41 metabolites of six groups of hormones were analyzed in and compared between Mu-legacy and ‘Legacy’ plants (Fig. 2, Table 1). Of the 14 gibberellins (GAs) that were measured, GA19 was detected in the leaf samples of both Mu-legacy and ‘Legacy’ plants, and the Mu-legacy leaves, compared with ‘Legacy’ leaves, showed much decreased GA19 (Fig. 2a). GA20 was detected high in the Mu-legacy sample [74.5 pmol/g dry weight (DW)] and low in two ‘Legacy’ samples (18.2 and 25.4 pmol/g DW, respectively) (Table 1). More importantly, a low level of GA4, which is an active GA form^36^, was present in two Mu-legacy samples but absent in all ‘Legacy’ samples (Table 1), indicating that the active GA4 was likely responsible for the promotion of flower bud formation as well as the nonchilled flower bud break (Fig. 1a). Seven ABA and ABA metabolites analyzed were all detected. The Mu-legacy leaves had a higher total ABA content (7.8 vs. 4.7 nmol/g DW) than the ‘Legacy’ leaves (Fig. 2b). Of the six auxin and auxin metabolites, only IAA was detected, and the Mu-legacy leaves showed a significantly (*p* = 0.01) lower content than the ‘Legacy’ leaves (Fig. 2c). The reduced IAA level likely contributed to the reduced size of the Mu-legacy plants (Fig. 1a). Of the ten cytokinin and cytokinin metabolites measured, four were detected in ‘Legacy’ and Mu-legacy samples; measurable isopentenyladenine (iP) was only found in the ‘Legacy’ leaves (Fig. 2d, Table 1). The overall levels of the detected cytokinin metabolites were higher in the ‘Legacy’ leaves (Fig. 2d). Salicylic acid (SA), jasmonic acid (JA), methyl jasmonate, and jasmonoyl isoleucine were measured and all detected in both the ‘Legacy’ and the Mu-legacy leaves. For the contents of JA and JA metabolites, there was no statistical difference between the ‘Legacy’ and Mu-legacy samples (Fig. 2e). The SA content was slightly higher in the Mu-legacy leaves, although not significant (Fig. 2f). These results showed that overexpressed *VcDDF1* and *VcRR2* induced content changes of some hormones in the Mu-legacy leaves.

**Table 1:** Concentrations of non-detected (n.d.) and low (<) quantitative hormones that show difference between the leaf tissues of ‘Legacy’ and Mu-legacy plants. DW: dry weight

Sample	Cytokinin (pmol/g, DW) iP	Gibberellins (pmol/g, DW)				
		GA4	GA7	GA8	GA20	GA53
Legacy sample 1	7.5	n.d.	<11.5*	<15.1*	18	n.d.
Legacy sample 2	5.6	n.d.	<11.5*	n.d.	25	<10.9*
Legacy sample 3	6.1	n.d.	<11.5*	<10.4*	<11.4*	n.d.
Mu-legacy sample 1	<4.9*	<24.7*	<12.1*	<11.0*	75	<11.5*
Mu-legacy sample 2	<4.4*	<19.9*	n.d.	n.d.	n.d.	n.d.
Mu-legacy sample 3	<4.9*	n.d.	<11.8*	n.d.	<11.7*	<11.2*

*p = 0.05

### Differentially expressed genes in Mu-legacy leaves

Previous comparisons of transcriptomic profiles of leaves between ‘Legacy’ and Mu-legacy plants have revealed that the increased expression of *VcRR2* likely co-functioned with the overexpressed *VcDDF1* for the promoted flowering and dwarfing of the Mu-legacy and Mu-legacy-T_1_ plants^25^. In this study, we identified 2543 DE transcripts (DETs) when transcriptomic profiles were compared between ‘Legacy’ and the Mu-legacy leaves and these DETs were much more than the 260 DETs identified in the previous transcriptomic comparison study [25]. Such a large difference likely reflects some variation in the biological samples (e.g., stages and sampling) and sequencing coverage. Nevertheless, among the 260 DETs identified from the previous study, 56 were overlapped with the DETs found in the current study. The enriched RNA-seq data not only verified the overexpressed *VcDDF1* and the increased expression of the *VcRR2*, but also provided more details to reveal the cause of the phenotypic changes in the Mu-legacy plant (Supplementary Tables 1 and 2).

**Table 2:** Differentially expressed transcripts (DETs) of the hormone and flowering genes identified from the comparison of leaf tissues of Mu-legacy and ‘Legacy’ plants

Query id	Subject id	Blast_e-value	Pathway	Gene name	Annotation by trinotate	Log_2_(Mu-legacy/Legacy) 2018 data	Log_2_(Mu-legacy/Legacy–VcDDF1-OX) 2014 data	Log_2_(Mu-legacy-T_1_/Legacy) 2014 data	Log_2_(Legacy–VcDDF1-OX/Legacy) 2014 data	Log_2_(Mu-legacy/Legacy) 2014 data
	c49456_g2_i2			VcARP6	ACTS_RAT	#N/A	−6.17	−4.73	#N/A	−5.98
	c66039_g1_i1			*npt*II	KKA2_KLEPN	10.29	#N/A	9.79	10.60	9.33
	c92529_g4_i2			VcRR2	ARR2_ARATH	8.08	5.77	6.15	#N/A	6.06
	c32575_g1_i1			VcDDF1	DRE1E_ARATH	10.28	−3.49	#N/A	7.64	5.31
	c96767_g2_i12			VcRR2	–	4.70	4.00	5.07	#N/A	#N/A
AT2G27690.1	c79501_g3_i1	4.00E−29	JA	CYP94C1	C96AF_ARATH	1.42	−2.34	3.16	#N/A	#N/A
AT1G75450.1	c83077_g1_i1	0	Cytokinin	ATCKX6, ATCKX5, CKX5	CKX5_ARATH	−1.62	2.00	#N/A	#N/A	#N/A
AT2G23620.1	c83935_g1_i1	6.00E−46	SA	MES1, ATMES1	MES17_ARATH	−0.97	1.18	−1.61	−0.82	#N/A
AT2G23620.1	c83935_g1_i2	2.00E−45	SA	MES1, ATMES1	MES17_ARATH	−0.79	1.26	−1.44	−0.90	#N/A
AT2G23620.1	c83935_g1_i3	8.00E−46	SA	MES1, ATMES1	MES17_ARATH	−0.75	1.27	−1.68	−0.82	#N/A
AT2G43820.1	c85921_g3_i2	2.00E−84	SA	GT, ATSAGT1, SGT1, UGT74F2, SAGT1	UGT1_GARJA	−0.68	1.34	#N/A	#N/A	#N/A
AT3G11480.1	c97521_g2_i1	2.00E−100	SA	BSMT1, ATBSMT1	–	−1.38	−3.54	#N/A	1.31	#N/A
AT4G25420.1	c89942_g2_i1	5.00E−55	GA	ATGA20OX1, AT2301, GA5, GA20OX1	SRG1_ARATH	−0.86	−1.37	#N/A	#N/A	#N/A
AT4G02780.1	c96791_g4_i1	0	GA	ATCPS1, CPS, CPS1, GA1, ABC33	KSA_PEA	1.60	2.89	2.31	#N/A	#N/A
AT1G13260.1	c83982_g1_i1	8.00E−132	Flower	RAV1, EDF4	RAV1_ARATH	1.35	3.07	−4.66	#N/A	#N/A
AT5G60910.1	c88116_g1_i1	5.00E−91	Flower	AGL8, FUL	AGL8_SOLTU	−2.38	4.23	#N/A	#N/A	#N/A
AT4G02050.1	c85501_g3_i2	8.00E−57	Sucrose	STP7	–	1.57	−1.62	1.52	#N/A	#N/A
AT4G21480.1	c85501_g3_i2	6.00E−54	Sucrose	STP12	–	1.57	−1.62	1.52	#N/A	#N/A
AT1G10370.1	c73618_g2_i1	1.00E−30	Sucrose	ATGSTU17, ERD9, GST30B, GST30, GSTU17	–	3.04	−1.97	3.23	1.66	#N/A
AT3G57040.1	c83921_g3_i2	3.00E−76		ARR9, ATRR4	ARR9_ARATH	−0.62	#N/A	#N/A	#N/A	#N/A
AT1G10470.1	c86949_g1_i2	5.00E−41		ARR4, MEE7, ATRR1, IBC7	ARR9_ARATH	−0.64	#N/A	#N/A	#N/A	#N/A

Corresponding DETs identified from the comparisons of leaf tissues between Mu-legacy and Legacy–VcDDF1-OX, Mu-legacy-T_1_ and ‘Legacy’, Legacy–VcDDF1-OX and ‘Legacy’, and Mu-legacy and ‘Legacy’ from a 2014 study were also included for the cross-comparison of the 2018 and 2014 data. #N/A: No differential expression

Only two DE genes (DEGs) of the flowering pathway, including orthologues of *Arabidopsis FRUITFULL* (*FUL*) and *Arabidopsis RAV1*, showed up- and down-regulation, respectively; both of which had repressive effects on flowering bud formation and flowering in the 2018 RNA-seq data. Although this result is inconsistent with the previously identified three DE flowering pathway genes (i.e., *VcTCP8*, *VcARP6*, and *VcNFYC1*)^25^, it still supports the previous conclusion that no orthologs of the major *Arabidopsis* vernalization pathway genes, e.g., *VcFT* and *VcSOC1*, showed differential expression. *RAV1* encodes an AP2/B3 domain and is a negative regulator of flowering by repressing both *FT* and GA^37^. If the function of the *RAV1* in *Arabidopsis* is conserved in blueberries, the upregulated *VcRAV1* would repress flowering of the Mu-legacy plants. *FUL* is redundant with *APETALA1* (*AP1*) and *CAULIFLOWER* (*CAL*) in promoting to the transition to reproductive meristems^38^. The decreased expression of the *VcFUL* would repress flower bud formation, which is inconsistent with the promoted flower bud formation of the Mu-legacy plants. Overall, the two DE flowering pathway genes were not likely responsible for the promoted flowering in the Mu-legacy plants.

**Fig. 3:**
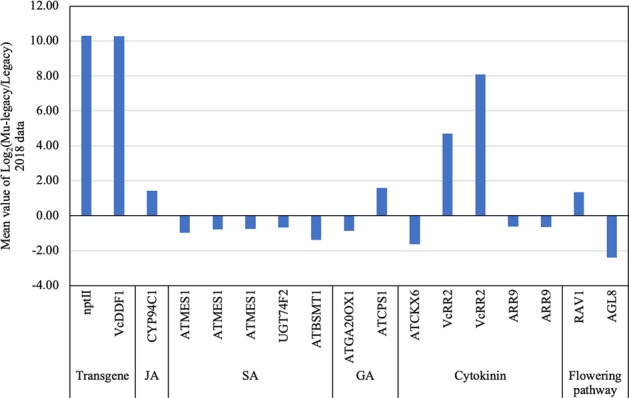
Differentially expressed transcripts (DETs) (PDR < 0.05) of the hormone and flowering genes identified from the comparison of leaf tissues of Mu-legacy and ‘Legacy’ plants in the current study (2018)

We identified seven DEGs related to the biosynthetic pathways of GA (2 DEGs), SA (3), JA (1), and cytokinin (1) but none for the ABA and auxin pathways (Table 2, Figs. 2, 3). *VcCPS* and *VcGA20OX1* were the two GA related genes. We found that *VcCPS* expression increased while *VcGA20OX1* expression decreased. *Ent*-COPALYL DIPHOSPHATE SYNTHASE (CPS) catalyzes the first step of GA biosynthesis^39^. The increased *VcCPS* could lead to an increase in bioactive GAs, this matched the result of the presence of the GA4 in the Mu-legacy leaves but not in the ‘Legacy’ leaves^36^. GA20OX1 catalyzes a series of intermediate oxidation reactions during the biosynthesis of GA^36^, the slightly reduced expression of *VcGA20OX1* should have reduced GA production. Whether the increased *VcCPS* and decreased *VcGA20OX1* expression were part of the cause for the reduced GA19 and increased GA4 content is unknown. CYTOKININOXIDASE6 (CKX6) catalyzes the degradation of cytokinins in *Arabidopsis*^40^, and we found that *VcCKX6* expressed reduced in this study. The reduced *VcCKX6* expression explained well the decreased content of cytokinins observed^41^. Similarly, we observed an increased expression of cytochrome P450 94C1 (CYP94C1), which is involved in the oxidation of jasmonoyl-L-isoleucine (JA-Ile) in *Arabidopsis*^42^. However, the increased *VcCYP94C1* did not lead to an increase of JA-Ile accumulation in this study. On the other hand, we found three SA-related DEGs in this study: *SALICYLATE/BENZOATE CARBOXYL METHYLTRANSFERASE (BSMT1)*, *SGT1*, and *METHYLESTERASE 17 (MES17)*. Decreased expressions of these genes were found to lead to accumulation of SA in *Arabidopsis*^43^, accordingly there was no surprise that the decreased *VcBSMT1*, *VcSGT1*, and *VcMES17* contributed to the increased SA in the Mu-legacy leaves. We observed the same association of increased expression of these genes (i.e., *VcBSMT1*, *VcSGT1*, and *VcMES17*) with increased accumulation of SA in the Mu-legacy leaves.

Because *VcRR2* could enhance cytokinin responses^35,44^, we searched for the DETs of the orthologues of the other ARR genes. Two DETs of *VcRR9* showed downregulation, which could potentially reduce cytokinin responses (Table 2, Fig. 3). Since cytokinins are often positively correlated to the IAA level^45^, the reduced cytokinin responses supported the reduced IAA content (Fig. 2c).

### The increased *VcRR2* expression promotes flower bud formation in Mu-legacy and Mu-legacy-T_1_ plants

A typical Legacy–VcDDF1-OX transgenic event, as expected, had the *VcDDF1* gene overexpressed in its leaves but did not show altered flowering behavior The *VcDDF1* gene was also overexpressed in the leaves of Mu-legacy Mu-legacy, which, however, was promoted to flower early. Interestingly, *VcRR2* and *VcCPS* were also overexpressed in Mu-legacy, but not in Legacy–VcDDF1-OX, suggesting that *VcRR2* and *VcCPS* were likely the causes for flowering promotion in Mu-legacy and had no direct connections with the overexpression of the *VcDDF1* gene in the background (Table 2). This conclusion was also confirmed in the Mu-legacy-T_1_ plants derived from self-pollinated Mu-legacy. The Mu-legacy-T_1_ plants showed a similar flowering behavior as the Mu-legacy. Very fortunately, in the DEGs between the ‘Legacy’ and Mu-legacy-T_1_ leaves, the *VcDDF1* did not showed differential expression but the *VcRR2* and the *VcCPS* were both upregulated (Table 2, Fig. 3). This suggests that the increased *VcRR2* and *VcCPS* promoted early flowering of the Mu-legacy-T_1_ plants under nonchilling conditions, most likely, through the gibberellin pathway genes.

### DE hormone genes are involved in dormancy release in nonchilled flower buds of Mu-legacy plants

We also examined the DETs in the flower buds from the Mu-legacy and ‘Legacy’ plants as these DETs might have direct impact on both flower bud development and dormancy release of the nonchilled flower buds (NB). We mainly focused on the DETs related to flowering and hormone genes. In addition to the highly increased *VcRR2* and four repressed DEGs in the flowering pathway [*PROTEIN FD (FD), TERMINAL FLOWER 1 (TFL1)*, *ACTIN-RELATED PROTEIN6* (*ARP6*), *DOF ZINC FINGER PROTEIN5.3* (*DOF5.3*) (*DOF5.3*)^25^, we identified 29 additional DETs, which are the orthologs of 17 *A. thaliana* genes. These genes belong to the biosynthesis pathway genes of five groups of hormones (Table 2, Fig. 3) and are likely involved in the dormancy release of the NB of the Mu-legacy plants.

Eight DETs representing three genes (*ABA2–4)* in the pathway of ABA synthesis and the *A. THALIANA BETA-GLUCOSIDASE 1* gene, which is a positive regulator of ABA-activated signaling pathway, were upregulated. These upregulated DETs could increase ABA content and enhance ABA signaling, thus potentially resulting in delaying flowering, which is contrary to the early flowering of the Mu-legacy (Table 3, Supplementary Table 2). Auxin interacts with ABA in controlling *A. thaliana* seed dormancy/germination^46^. Eight DETs of four auxin biosynthetic genes exhibited a potential for an increase in auxin biosynthesis (Table 3). Brassinosteroids (BRs) play a critical opposite role to ABA during seed germination and deficiency of BRs is often associated with dwarf phenotypes^47^. Five DETs of three genes in the pathway of BRs biosynthesis, including four upregulated and one downregulated DETs (Table 3), most likely inactivate BRs^48,49^. The reduced active BRs, working with others, can consequently lead to the dwarfing and delayed flowering in *A. thaliana*^50^. Cytokinins are multifunctional in plants. Four DETs representing three DE cytokinin genes similar to the *CYP735A1*, *UGT85A1*, and *APT5* genes in *Arabidopsis* were identified (Table 3), the overall impact of the DETs can lead to cytokinin-deficient plants due to their potential to reduce active cytokinins through enhanced O-glucosylation^51–53^. JA is involved in chilling-dependent dormancy release through its interaction with ABA^54^. Four DETs of four genes in the JA biosynthetic pathway were identified (Table 3), and they all showed an upregulation that could either increase or reduce the production of JA^55,56^. It is interesting that unlike in the leaf tissues we did not detect any DEGs of the GA pathway in the transcriptome comparison between the nonchilled buds of the Mu-legacy and the ‘Legacy’, suggesting that the promoted flowering of the nonchilled Mu-legacy buds had little to do with the expression the GA pathway genes in this case. Apparently, no convincing evidence shows that any of the individual hormone(s) or the DEG of these hormones is responsible for the promoted flowering of the no-chilled Mu-legacy buds.

**Table 3:** Differentially expressed transcripts (DETs) of hormone genes identified in the transcriptome comparison between nonchilled flower bud (NB) of the Mu-legacy and ‘Legacy’ plants (2014 data)

Query id	Subject id	Blast_e-value	Pathway	Gene name	Annotation by Trinotate	Legacy		Mu-legacy		Log_2_(Mu-legacy NB/ Legacy NB)
						Log_2_(CB/NB)	Log_2_(CB/LPB)	Log_2_(CB/NB)	Log_2_(CB/LPB)	
	c49456_g2_i2			VcARP6	ACTS_RAT	−8.91	−9.43	#N/A	−5.55	6.89
	c66039_g1_i1			*npt*II	KKA2_KLEPN	#N/A	#N/A	#N/A	#N/A	−8.57
	c92529_g4_i2			VcRR2	ARR2_ARATH	#N/A	#N/A	#N/A	#N/A	−7.26
	c32575_g1_i1			VcDDF1	DRE1E_ARATH	−2.23	−4.84	#N/A	#N/A	−6.75
	c96767_g2_i12			VcRR2	–	#N/A	#N/A	#N/A	#N/A	−4.22
AT5G45340.1	c69424_g1_i1	2.00E−65	ABA	CYP707A3	ABAH3_ORYSI	6.64	5.88	5.16	6.66	−5.37
AT3G19270.1	c69424_g2_i1	5.00E−124	ABA	CYP707A4	ABAH4_ARATH	5.66	7.72	5.16	10.15	−5.08
AT5G45340.1	c69424_g2_i1	8.00E−100	ABA	CYP707A3	ABAH4_ARATH	5.66	7.72	5.16	10.15	−5.08
AT3G19270.1	c83950_g1_i2	0	ABA	CYP707A4	ABAH4_ARATH	6.62	−1.53	4.74	#N/A	−4.77
AT2G29090.1	c83950_g1_i1	0	ABA	CYP707A2	–	6.41	−1.38	4.74	#N/A	−4.60
AT1G52400.1	c91881_g1_i1	5.00E−89	ABA	BGL1, ATBG1, BGLU18	HIUH_SOYBN	1.94	−0.49	#N/A	−2.51	−1.67
AT1G52400.1	c80508_g1_i1	9.00E−134	ABA	BGL1, ATBG1, BGLU18	RG1_RAUSE	#N/A	#N/A	#N/A	−1.84	−1.61
AT1G52400.1	c91881_g1_i2	7.00E−82	ABA	BGL1, ATBG1, BGLU18	HIUH_SOYBN	1.38	#N/A	#N/A	−2.03	−1.18
AT2G22330.1	c85230_g2_i1	9.00E−56	Auxin	CYP79B3	C70A2_ARATH	11.98	11.82	0.50	10.93	−7.88
AT4G31500.1	c94863_g6_i2	1.00E−62	Auxin	RNT1, RED1, SUR2, ATR4, CYP83B1	C81E8_MEDTR	2.75	#N/A	#N/A	−2.87	−1.85
AT4G31500.1	c93443_g1_i1	2.00E−65	Auxin	RNT1, RED1, SUR2, ATR4, CYP83B1	C81E8_MEDTR	2.06	2.53	#N/A	−1.19	−1.40
AT4G31500.1	c93443_g1_i3	1.00E−65	Auxin	RNT1, RED1, SUR2, ATR4, CYP83B1	C81E8_MEDTR	2.32	3.12	#N/A	−1.59	−1.20
AT1G08980.1	c89856_g5_i1	7.00E−163	Auxin	ATAMI1, AMI1, TOC64-I, ATTOC64-I	AMI1_ARATH	#N/A	#N/A	#N/A	0.66	0.66
AT4G31500.1	c83219_g1_i2	4.00E−43	Auxin	RNT1, RED1, SUR2, ATR4, CYP83B1	C78A5_ARATH	−1.36	4.08	0.54	2.46	1.05
AT2G22330.1	c90066_g1_i1	2.00E−47	Auxin	CYP79B3	C82A3_SOYBN	0.95	4.97	0.56	#N/A	1.45
AT5G11320.1	c87553_g1_i1	7.00E−85	Auxin	AtYUC4, YUC4	YUC4_ARATH	−1.69	5.66	#N/A	−1.54	1.91
AT2G36800.1	c93075_g2_i1	6.00E−88	BR	UGT73C5, DOGT1	–	5.01	#N/A	#N/A	2.32	−1.80
AT2G26710.1	c97437_g3_i3	1.00E−123	BR	CYP734 A1, CYP72B1, BAS1	C7A22_PANGI	1.85	#N/A	#N/A	−1.87	−1.42
AT1G17060.1	c93255_g4_i4	8.00E−74	BR	CHI2, SOB7, CYP72C1, SHK1	C7A29_PANGI	#N/A	2.51	#N/A	−1.49	−1.30
AT2G26710.1	c93255_g4_i5	9.00E−140	BR	CYP734 A1, CYP72B1, BAS1	C7A29_PANGI	#N/A	2.74	#N/A	−1.37	−1.02
AT2G26710.1	c54735_g3_i1	6.00E−112	BR	CYP734A1, CYP72B1, BAS1	C72A1_CATRO	−2.01	−7.66	6.67	−7.85	3.35
AT1G22400.1	c92329_g1_i1	2.00E−52	Cytokinin	UGT85A1, ATUGT85A1	UFOG_VITVI	5.36	#N/A	#N/A	1.15	−3.31
AT2G36800.1	c93075_g2_i1	6.00E−88	Cytokinin	UGT73C5, DOGT1	–	5.01	#N/A	#N/A	2.32	−1.80
AT5G11160.1	c82929_g1_i1	5.00E−108	Cytokinin	APT5	APT5_ARATH	#N/A	#N/A	#N/A	0.74	0.77
AT5G11160.1	c82929_g1_i2	2.00E−109	Cytokinin	APT5	APT5_ARATH	#N/A	#N/A	#N/A	#N/A	1.28
AT1G20510.1	c79869_g1_i1	4.00E−103	JA	OPCL1	4 CLL1_ARATH	12.22	11.44	#N/A	11.67	−7.33
AT3G48520.1	c93124_g4_i1	3.00E−95	JA	CYP94B3	C70B1_ARATH	9.17	4.24	#N/A	5.56	−5.99
AT1G44350.1	c91828_g2_i1	0	JA	ILL6	ILL6_ARATH	2.55	#N/A	−0.50	−3.50	−1.31
AT5G07010.1	c90152_g5_i1	4.00E−86	JA	ST2A	SOT15_ARATH	1.09	1.27	#N/A	−1.66	−0.94

Corresponding DETs identified from the other four transcriptome comparison of the flower bud tissues, including chilled (CB) vs. NB and chilled vs. late-pink bud (LPB) for ‘Legacy’ and Mu-legacy plants, respectively, were also included. #N/A: No differential expression

Since hormone genes are involved in blueberry flower bud dormancy release^22^, we further looked into the expression of the 29 DETs of 17 hormone genes identified in the NB of Mu-legacy plants in CB and late-pink buds (LPB) of both Mu-legacy and ‘Legacy’ plants (Table 3). In the comparison of CB and NB of ‘Legacy’, 23 transcripts showed differential expressions, of which 22 showed a consistent up- or down-regulation with those in the 29 DETs resulting from the comparison between NB of Mu-legacy and ‘Legacy’ (Table 3), supporting that the 29 DETs are responsible for the promoted flowering of the “Mu-legacy” plants under nonchilling conditions. In the comparison of CB and LPB of ‘Legacy’, 18 of the 29 transcripts exhibited differential expression during flower bud break (Table 3). On the other hand, according to the low percentage of bud break and the reduced number of flowers per bud^25^, the 29 DETs did not initiate the normal flowering of nonchilled Mu-legacy plants, most likely because more chilling-driven DETs of other hormone genes or the flowering pathway genes are needed to induce normal flowering (Supplementary Table 2). In addition, two comparisons for Mu-legacy plants, including chilled buds versus nonchilled buds and LPB of the Mu-legacy plants, respectively, revealed 19 and 24 DETs of the 29 transcripts of 17 hormone genes were inducers of the Mu-legacy plant flowering under chilling conditions (Table 3). This provides further evidence that the 29 DETs are responsible for flowering of the “Mu-legacy” plants under nonchilling conditions. The 17 DETs identified in the comparison of chilled ‘Legacy’ buds and nonchilled “Mu-legacy” buds indicated that insufficient changes of these transcripts could also be responsible for the unusual flowering behaviors of the nonchilled Mu-legacy plants.

### DE flowering pathway genes are involved in dormancy release in fully chilled flower buds of Mu-legacy plants

Bud break of the CB of the Mu-legacy plants showed no obvious difference from that of the ‘Legacy’ plants, and the flowering time lasted about 2 weeks^25^. To look into the effect of fully chilling on flowering of the Mu-legacy plants at transcript levels, we compared transcriptome data of the CB with those of NB and LPB, respectively. In both comparisons, DE flowering pathway genes were identified, including 455 DETs in the comparison of the CB vs. the NB and 396 DETs in the comparison of the CB vs. the LPB (Supplementary Table 3). For the ‘Legacy’ plants, the comparison of the CB vs. the NB resulted in 413 DETs of the flowering pathway genes, of which 304 are shared with the 455 DETs of the “Mu-legacy” plants. Of the shared 304 DETs, 302 showed the same upregulations or downregulations. The comparison of the CB vs. the LPB of the ‘Legacy’ plants resulted in 642 DETs of the flowering pathway genes, in which 299 is shared in the 396 DETs of the “Mu-legacy” plants. Of the shared 299 DETs, 285 showed the same upregulations or downregulations (Supplementary Table 3).

Only six DETs of four flowering pathway genes, including *VcFD, VcTFL1*, *VcARP6*, and *VcDOF5.3*, were detected and were all repressed in the NB of the Mu-legacy plants (compared with the nonchilled ‘Legacy’ buds)^25^. We analyzed expression of the six transcripts in the fully vernalized flower buds and the LPB of the Mu-legacy plants. None of the six transcripts showed differential expressions in the comparison of the CB with those of nonchilled CB. In contrast, five of the six transcripts showed differential expressions in the comparison of the CB with the LPB, of which expression of the *VcARP6* was repressed and expression of the other three genes were upregulated (Supplementary Table 4). This indicates that the four genes were involved in flowering of the CB of the Mu-legacy plants. In the ‘Legacy’ plants, we further looked into expression of the six transcripts in both the CB and the LPB. Five transcripts of the four genes were all repressed in the CB (compared with the NB). The result was similar to the six DETs identified in the comparison between the NB of the Mu-legacy plants and the NB of the ‘Legacy’ plants (Supplementary Table 4). This suggests that the four genes were chilling responsive in the ‘Legacy’ flower buds and their expression changes in the NB of the Mu-legacy plants were most likely responsible, at least in part, for the flowering of the nonchilled Mu-legacy plants. All of the six transcripts were differentially expressed in the LPB (compared with the CB) of the ‘Legacy’ plants; the result is similar to that of the comparison of the CB with those of LPB of the Mu-legacy plants (Supplementary Table 4).

### DE hormone genes are involved in dormancy release in fully chilled flower buds of Mu-legacy plants

In the ‘Legacy’ plants, 703 and 804 DETs of the genes of four hormones (i.e., ABA, ethylene, auxin, and GA) and ARR genes, were identified in CB (compared with NB) and LPB (compared with CB), respectively^22^. In the Mu-legacy plants, 565 and 276 DETs of nine groups of hormone genes were identified in CB (compared with NB) and LPB (compared with CB), respectively (Supplementary Table 2).

## Discussion

### Cytokinins and gibberellins promote flower bud formation in the Mu-legacy plants

Leaves are the primary sources of florigen signals in inducing flowering^57^. For blueberries, overexpression of *VcFT* (*VcFT*-OX) is able to stimulate continuous flower bud formation of transgenic “Aurora” through both the DE flowering pathway genes and the DE hormone genes (compared with nontransgenic leaves)^23,24,58^, which was associated with changes in the contents of different hormones (unpublished data). Apparently, expression of hormone genes and hormones abundances are responsive to the *VcFT*-OX, although it is impossible to conclude whether or not these hormones are part of the florigenic signals because of the well-documented leaves-to-buds transport of FT proteins in many herbaceous plants^59–67^. In another case, *SOC1* is a major integrator downstream of *FT*^10^. Overexpression of the K-domain of the *VcSOC1* (*VcSOC1K*-OX) in “Aurora” leaves enhanced flower bud formation through a group of DE hormone genes and DE flowering pathway genes (compared with nontransgenic leaves) where the *VcFT* did not show differential expression^26^. Thus, the promoted flower bud formation is not likely, at least at transcript levels, entirely through the enhanced VcFT production and transport. In fact, phenotypic changes in both the *VcFT*-OX and the *VcSOC1K*-OX plants suggest that hormones could have played essential roles in flower bud formation and dormancy release^23,26^.

The Mu-legacy plants are phenotypically similar to both *VcFT*-OX and *VcSOC1K*-OX plants and showed promoted flower bud formation and flowering under nonchilling conditions^25^. However, the major difference is that only two DE flowering pathway genes (compared with nontransgenic ‘Legacy’ leaves), in contrast to the many more identified in the *VcFT*-OX and the *VcSOC1K*-OX plants^24,26^, were detected. More interestingly, the two DE flowering pathway genes, including the upregulated *VcRAV1* and the downregulated *VcFUL* (Table 2, Fig. 3), have repressive effects on flowering bud formation and flowering. This provides evidence that the signals promoting flower bud formation in the Mu-legacy leaves are independent of expression of *VcFT* and flowering pathway genes. Therefore, we believe that the seven DEGs related to biosynthetic pathways of GA, SA, JA and cytokinin, especially the contents of cytokinins and GAs, were most likely responsible for the promoted flower bud formation in the Mu-legacy plants (Table 2, Figs. 2, 3).

### Responses of blueberry flowering pathway genes during dormancy release

Sufficient chilling hours and warm temperatures are two prerequisites to break blueberry dormancy for healthy flowering and growth^4^. Accordingly, blueberry flower buds often experience two major changes of their physiological statuses, including transition from nonchilled to chilled buds following full chilling and then from chilled buds to flowers upon exposure to warmer temperatures. In the previous transcriptome comparisons for ‘Legacy’ plants, the comparison of chilled and NB resulted in differential expression of 89% of the blueberry flowering pathway genes; and, the comparison of CB and late-pink flower revealed differential expression of 96% of flowering pathway genes^22^. Apparently, flowering pathway genes are highly involved in dormancy release of blueberry buds at transcript levels. Interestingly, only four (3.8%) flower pathway genes, including *VcFD, VcTFL1*, *VcARP6*, and *VcDOF5.3*, showed differential expression in the nonchilled buds of Mu-legacy, of which ~50% were able to flower under nonchilling conditions^25^. Although this does not look reasonable for the Mu-legacy plants at transcript levels, it is possible at post-transcriptional or translational levels because little is known about what physiological signals are actually induced by these DE flowering pathway genes identified in dormancy release. One fact is that many DE hormone genes were also induced in ‘Legacy’ flower buds during dormancy release^22^. It remains unclear whether the DE flowering pathway genes or DE hormone genes determine the dormancy release. Thus, the value of the Mu-legacy flowering under nonchilling conditions associated with only 4 DE flowering pathway genes is that it demonstrates that other pathway genes, e.g., the 29 DE transcripts of 17 hormone genes (Table 3), play a significant role in blueberry flower bud break.

### Mechanism of chilling-mediated flowering of the Mu-legacy plants

**Fig. 4:**
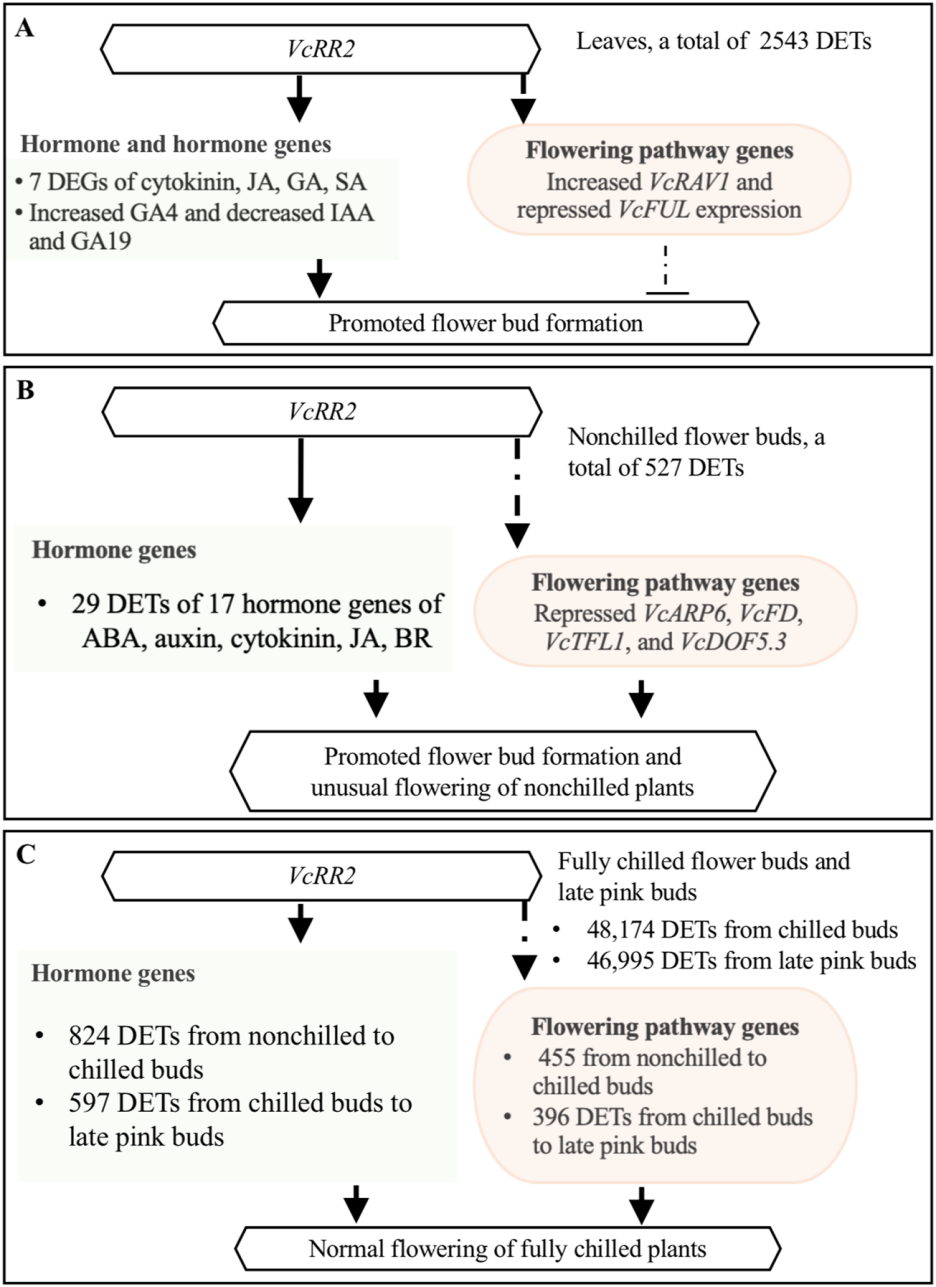
Schematic diagram illustrating the effect of the enhanced expression of the *VcRR2* on flower bud formation and flowering of the Mu-legacy plants. **a** The differential expressed transcripts (DETs) of hormone genes (compared with ‘Legacy’) and the changed contents of GAs in leaves could be responsible for the promoted flower bud formation. **b** In total, 29 DETs of 17 hormone genes played a major role in stimulating the flower bud break and unusual flowering of the Mu-legacy plants under nonchilling conditions. **c** Normal flowering of the Mu-legacy plants after full chilling was driven by the DETs of both hormone and flowering pathway genes

Mechanisms underlying release of bud endodormancy of deciduous trees are complicated due to the involvement and interaction of multiple pathways^68,69^. Hormone and flowering pathways are involved in the dormancy release of blueberry flower buds^22^. The Mu-legacy plants showed early flower bud formation in 1- and 2-year-old plants when few flower buds were observed in the nontransgenic ‘Legacy’ plants^25^. In addition, for the plants of the same age, the Mu-legacy plants had more flower buds than the nontransgenic ‘Legacy’ plants^25^. Only two DEGs of the flowering pathway genes, including zero in the previous comparison^25^ and two in this study, were detected in the two transcriptome comparisons between the leaf tissue of the Mu-legacy plants and that of the nontransgenic ‘Legacy’ plants. It was likely that the DE flowering pathway genes played a minor role in the promoted Mu-legacy flowering. Accordingly, if the florigenic signals for the promoted flower bud formation in the Mu-legacy plants were from leaves, the DETs of hormone genes were more likely the major cause than the DETs of the flowering pathway genes (Fig. 4a). Based on the transcriptome data and the hormone profile in the leaves of the Mu-legacy plants, we believe that it was the expression of *VcRR2* that enhanced plant response to cytokinins, which consequently promoted flower bud formation through the changes of either the content of hormones (e.g., GA4) or the expression of the hormone genes (e.g., *VcCPS*) (Table 2, Fig. 4a).

In the NB of the Mu-legacy plants, only four DEGs of the flowering pathway genes, including *VcFD, VcTFL1*, *VcARP6*, and *VcDOF5.3*, were detected and were all repressed (compared with NB of the nontransgenic ‘Legacy’ plants)^25^. In addition, 29 DETs of 17 hormone genes of ABA, auxin, cytokinin, JA, and BR were found (Table 3). These 4 DEGs flowering pathway genes 17 DEGs of hormone genes were associated with mainly by the expression of *VcRR2* and resulted in unusual flowering of the NB (Fig. 4b).

From nonchilled to CB and to LPB of both the ‘Legacy’ and the Mu-legacy plants, a great number of flowering pathway genes and hormone genes were involved (Supplementary Tables 2 and 3). For fully chilled plants, the interactions of these genes under warm conditions drove normal bud break and flowering (Fig. 4c).

## Materials and methods

### Plant materials

Six 5-year-old plants for each of nontransgenic highbush blueberry ‘Legacy’ and transgenic Mu-legacy and 12 plants of three Legacy–VcDDF1-OX transgenic lines were grown in the courtyard between two greenhouses for phenotyping in 2017. The plants were moved into the greenhouse (heated for winter) in the October of 2017. In January of 2018, young leaf tissues, 5–10 g for each of the ‘Legacy’ and Mu-legacy plants, were harvested from multiple new shoots; half of these leaves were subjected to freeze drying immediately and another half were ground in liquid nitrogen and stored in a −80 °C freezer. Six Mu-legacy-T_1_ plants, which are from one self-pollinated T_1_ seed of Mu-legacy^25^, were grown for phenotyping in the greenhouse since 2016 and never exposed to chilling temperatures. All plants were developed from in vitro cultured shoots and grown under natural light conditions and a regular schedule of irrigation and fertilization using 0.2 g/L fertilizer (Nitrogen:Phosphorus:Potassium = 21:7:7)^70^.

### Hormone profiling

Freeze-dried tissues of three plants each from ‘Legacy’ and Mu-legacy plants were used for profiling ABA and six ABA metabolites (ABAGE: Abscisic acid glucose ester, DPA: Dihydrophaseic acid, PA: Phaseic acid, 7′OH-ABA: 7′-Hydroxy-abscisic acid, neo-PA: *neo*-Phaseic acid, and *t*-ABA: *trans*-Abscisic acid), auxins [IAA: Indole-3-acetic acid, IAA-Asp: N-(Indole-3-yl-acetyl)-aspartic acid, IAA-Glu: N-(Indole-3-yl-acetyl)-glutamic acid, IAA-Ala: N-(Indole-3-yl-acetyl)-alanine, IAA-Leu: N-(Indole-3-yl-acetyl)-leucine, and IBA: Indole-3-butyric acid], cytokinins [*t*-ZOG: (*trans*) Zeatin-O-glucoside, *c*-ZOG: (*cis*) Zeatin-O-glucoside, *t*-Z: (*trans*) Zeatin, *c*-Z: (*cis*) Zeatin; dhZ: Dihydrozeatin, *t*-ZR: (*trans*) Zeatin riboside, *c*-ZR: (*cis*) Zeatin riboside, dhZR: Dihydrozeatin riboside, iP: Isopentenyladenine, and iPR: Isopentenyladenosine], and 14 gibberellins (GA1, GA3, GA4, GA7, GA8, GA9, GA19, GA20, GA24, GA29, GA34, GA44, GA51, and GA53). These hormones were measured by the National Research Council of Canada, Saskatoon, SK S7N 0W9 (http://www.nrc-cnrc.gc.ca/eng/solutions/advisory/plant_hormone.html). Fresh tissues of the same set of materials for RNA-seq and other hormone analysis were used for quantification of SA, JA, and two JA metabolites (MeJA: Methyl jasmonate, JA-Ile: jasmonoyl isoleucine). The samples were prepared following an *Arabidopsis* protocol^71^ and measured by the Mass Spectrometry and Metabolomics Core of Michigan State University. ANOVA and Tukey’s test were conducted using RStudio (Version 1.0.136).

### RNA-seq and differential expression analysis

Total RNA of the same set of samples used in hormone analysis was each isolated from ~0.5 g of ground tissues using a CTAB method^72^, followed by using the RNeasy Mini Kit for on-column DNase digestion and RNA purification (Qiagen, Valencia, CA, USA). The integrity of the RNA samples was assessed using the Agilent RNA 6000 Pico Kit (Agilent Technologies, Inc., Germany). All RNA samples submitted for RNA sequencing had an RNA quality score >8.0. Sequencing (150-bp pair-end reads) was conducted using the Illumina HiSeq4000 platform at the Research Technology Support Facility at Michigan State University (East Lansing, MI, USA). In total, 30–60 million reads were generated for each biological replicate. The FastQC program (www.bioinformatics.babraham.ac.uk/projects/fastqc/) was used to assess the quality of sequencing reads, with the per base quality scores ranging from 30 to 40.

The RNA-seq reads were analyzed using Trinity^73^ and aligned to the transcriptome reference Reftrinity (GenBank accession number: SRX2728597)^24^, and the abundance for each of a single read was estimated using the Trinity command “align_and_estimate_abundance.pl”. The Trinity command “run_DE_analysis.pl–method edgeR” was used to conduct a differential expression analysis^73^. The differentially expressed (DE) genes or isoforms with ta false discovery rate (FDR) value below 0.05 (*p-*value < 0.001) were used for further pathway analysis. Fragments per kilobase of transcript per million mapped reads (FPKM) were used to evaluate expression abundance. Most of the analyses were performed using the resources at the High Performance Computing Center of Michigan State University.

We focused our analyses on the genes of three pathways: hormone, sugar, and flowering. Synthesis pathway genes of nine phytohormones in *Arabidopsis*, including auxin, cytokinin, ABA, ethylene, gibberellin, brassinosteroid, jasmonic acid, salicylic acid, and strigolactones, were retrieved from the RIKEN Plant Hormone Research Network (http://hormones.psc.riken.jp/). Similarly, sugar synthesis pathway genes in *Arabidopsis* were used as references in analyzing sugar-related genes in this study (Supplementary Table 5). All of the selected *Arabidopsis* genes were used as queries to blast against the transcriptome reference Reftrinity^24^ and the blueberry isoforms showing e-values less than e^−20^ were used for further various transcriptome comparisons. On the other hand, blueberry flowering pathway genes identified in our previous study^24^ were referenced for analyzing the DETs related to flowering pathways in this study.

Some additional transcriptome comparisons were carried out by using data from our previous reports^22,25,74^, as listed in Supplementary Table 6. The previous transcriptome data involved in the comparisons had three biological replicates for each sample and an RNA quality score above 8.0. The data were collected by sequencing 100-bp pair-end reads using the Illumina HiSeq2500 platform at the Research Technology Support Facility at Michigan State University (East Lansing, MI, USA). All the raw sequencing data had been deposited in GenBank.

## Supplementary information

Supplementary Table 1

Supplementary Table 2

Supplementary Table 3

Supplementary Table 4

Supplementary Table 5

Supplementary Table 6
