# Supplementary material for: VcRR2 regulates chilling-mediated flowering through expression of hormone genes in a transgenic blueberry mutant
Source: Hortic Res. 2019 Aug 21;6:96. doi: 10.1038/s41438-019-0180-0 (PMC6804727; doi:10.1038/s41438-019-0180-0)
Supplement: Supplementary file 4 — Supplementary Table 4 [file 41438_2019_180_MOESM4_ESM.docx]

**Table S4** Differentially expressed flowering pathway genes in chilled flower buds (CB), nonchilled flower buds (NB), and late pink buds (LPB) of Mu-legacy and ‘Legacy’ plants. The flowering pathway genes of blueberry are orthologues of the flowering pathway genes of *A. thaliana*, rice, and cereals (e <-20) ^17^. #N/A: No differential expression

| Transcript_id | Log_2_(Legacy CB/Legacy NB) | (Legacy CB/ Legacy LPB) | Log_2_(Mu-Legacy NB/ Legacy NB) | Log_2_(Mu-Legacy CB/ Mu-Legacy NB) | Log_2_(Mu-Legacy CB/ Mu-Legacy LPB) | Annotation by Trinotate |
| --- | --- | --- | --- | --- | --- | --- |
| c49456_g2_i1 | -7.69 | -8.08 | -6.32 | #N/A | -5.66 | ACTS_RAT, ARP6 |
| c49456_g2_i2 | -8.91 | -9.43 | -6.89 | #N/A | -5.55 | ACTS_RAT, ARP6 |
| c22179_g1_i1 | -2.33 | 3.75 | -2.55 | #N/A | 2.59 | CET2_TOBAC, TFL1 |
| c98207_g4_i2 | -0.55 | 1.75 | -0.75 | #N/A | #N/A | DOF53_ARATH |
| c98207_g4_i1 | #N/A | 1.91 | -0.71 | #N/A | 0.77 | DOF53_ARATH |
| c75407_g1_i2 | -2.96 | 4.49 | -2.41 | #N/A | 3.67 | FD_ARATH |
